# Influences of footstrike patterns and overground conditions on lower extremity kinematics and kinetics during running: Statistical parametric mapping analysis

**DOI:** 10.1371/journal.pone.0317853

**Published:** 2025-02-06

**Authors:** Yifang Zhuang, Wenxing Zhou, Ziwei Zeng, Shiwei Mo, Lin Wang

**Affiliations:** 1 School of Exercise and Health, Shanghai University of Sport, Shanghai, China; 2 Department of Sports Science and Physical Education, The Chinese University of Hong Kong (CUHK), Hong Kong, China; 3 Division of Sports Science and Physical Education, Shenzhen University, Shenzhen, Guangdong, China; University of Essex, UNITED KINGDOM OF GREAT BRITAIN AND NORTHERN IRELAND

## Abstract

This study investigated lower extremity biomechanics when running on different surfaces among runners with different footstrike patterns. Thirty rearfoot strikers (RFSs) and non-rearfoot strikers (nRFSs) ran at 3.3 m/s on a specially designed indoor track covered with three surfaces: artificial grass, synthetic rubber, and concrete. A motion capture system with ten cameras combined a force plate was used to collect marker trajectory and ground reaction force (GRF) during the running stance phase. A two-way analysis of variance with statistical parametric mapping was employed to evaluate differences in the biomechanics of the lower extremities between footstrike patterns and among running surfaces. The nRFSs exhibited significantly greater ankle inversion angles and increased inversion and internal rotation moments at midstance compared to the RFSs. Conversely, the RFSs demonstrated significantly greater knee abduction moments in late stance. Running on stiffer surfaces was associated with greater vertical GRF in late stance, as well as increased knee and hip extension moments during midstance. Furthermore, running on stiffer surfaces was associated with increased knee abduction moments, hip abduction moments, and hip external rotation moments during late stance. These findings suggested that nRFSs endure more ankle loads, while RFSs face increased knee loads. However, regardless of the footstrike pattern, runners may benefit from selecting softer surfaces to reduce the risk of injury.

## Introduction

Running is a widely embraced sport renowned for fostering physical and mental well-being [1, 2]. However, between 19.4% and 79% of runners experience sports injuries during running, and 97% of these injuries are located in lower extremities [3, 4]. The risk factors for running injuries are multifaceted [5], encompassing both external factors, such as running shoes [6] and surface conditions [7–9], as well as internal factors like footstrike patterns [10] and lower extremity biomechanical abnormalities [11]. Numerous studies have indicated that footstrike patterns and running surface conditions may contribute to the incidence of lower extremity injuries during running [7, 12, 13].

The variety of surfaces encountered during running typically comprise synthetic rubber, concrete, asphalt, artificial grass, and woodchip surfaces; all of which exhibit varying degrees of stiffness, elasticity, and/or smoothness, thereby influencing running biomechanics [7, 8, 11, 14]. For rearfoot strikers (RFSs), running on stiff surfaces is associated with greater ankle and knee flexion angles compared to running on softer surfaces [8, 15, 16]. Studies have shown that, compared to soft surfaces, RFSs running on stiffer surfaces exhibited higher ground reaction forces (GRFs) [16] and increased plantar pressure [7]. Furthermore, wearable sensors have been used to examine the effects of surface conditions during running. Boey et al. [17] found that vertical acceleration measured on the tibia was higher when running on stiff surfaces (i.e., concrete and synthetic track) than on soft surfaces (i.e., woodchip surfaces). This finding is consistent with studies that link GRFs and loading rates to tibial vertical acceleration [18–20]. Research has shown that running on softer surfaces is associated with lower peak GRFs [16] and lower peak pressures [21] compared to stiffer surfaces. This may suggest that running on softer surfaces could mitigate the risk of leg injuries, such as tibial stress fractures. However, it is important to note that these studies did not differentiate between the footstrike patterns of the runners.

Our research group has previously investigated the biomechanical characteristics of lower extremities during running on different overground surfaces among habitual non-rearfoot strikers (nRFSs) using the same study sample cohort [13, 22, 23]. Existing studies indicate that nRFSs experience reduced forefoot plantar loads when running on soft surfaces (e.g., synthetic rubber or artificial grass) [23]. However, this adaptation is accompanied by increased ankle joint loading [22] and elevated GRFs during running [13]. Zhou et al. [13] found that nRFSs exhibit lower hip, knee, and ankle joint moments when running on synthetic rubber compared to stiffer surfaces (e.g., concrete), suggesting that this surface my help attenuate lower extremity joint loads. To the best of our knowledge, few studies have specifically recruited RFSs, but it has been found that running on softer surfaces, such as natural grass, can reduce in-shoe plantar pressure in recreational runners [7, 24], thereby decreasing the risk of injury during running [24]. Nevertheless, current investigations predominantly recruit runners with singular habitual footstrike pattern [7, 13, 16, 22, 23] or do not distinguish between different footstrike patterns among recreational runners [25, 26]. Therefore, no studies have simultaneously recruited runners with different footstrike patterns to test various surface conditions, aiming to provide comprehensive insights into the combined effects of these two variables on lower extremity biomechanics.

Running footstrike patterns strongly influence the lower extremity biomechanics of runners [27–31]. Compared to RFSs, nRFSs exhibited greater ankle dorsiflexion angles during running [27, 28] and increased internal rotation angles at the knee joint [29]. In terms of kinetics, Thompson et al. [29] reported that nRFSs exhibited lower peak GRFs. Additionally, studies by Kulmala et al. [30] and Valenzuela et al. [31] observed that nRFSs experienced higher peak knee adduction moments. Our preliminary investigations revealed that RFSs exhibited elevated total midfoot force and a higher foot-to-toe index, with predominant plantar loads concentrated in the rearfoot and midfoot regions [12]. This biomechanical pattern may heighten the susceptibility of RFSs to patellofemoral pain, as they bypass the ankle absorption phase during landing, leading to greater impact transfer to the knee joint compared to nRFS runners [12]. Although nRFSs demonstrate lower overall plantar loads, their increased forefoot loading may predispose them to metatarsal stress fractures and potential compensatory injuries to the Achilles tendon and triceps surae [32, 33].

Previous studies have primarily focused on the influence of singular footstrike pattern on running biomechanics across various surfaces and the lower extremity biomechanics associated with different footstrike patterns. However, research simultaneously addressing the running biomechanics of both footstrike patterns across different surfaces remains limited. More comprehensive studies are needed to provide tailored surface selection recommendations for runners with different footstrike patterns. Statistical parametric mapping (SPM) analysis, which enables comparison of biomechanical data across the entire gait cycle or phase, offers an effective complement to traditional discrete analyses by allowing for the visualization of continuous, time-varying biomechanical patterns [34]. For example, SPM can reveal subtle variations in joint angles or forces throughout the entire stance phase, which may not be captured in discrete analysis methods that focus on specific events such as heel strike or toe-off. Despite its utility, SPM has not yet been applied to investigate the biomechanics of different footstrike patterns on varied running surfaces.

The purpose of this study was to examine the effects of three overground surfaces and two habitual footstrike patterns on running biomechanics. We aimed to provide evidence-based recommendations on optimal surface selection for runners with different footstrike patterns to minimize the risk of running-related injuries. The hypotheses were as follows: (1) running on a stiffer surface would result in increased GRFs and ankle joint moments, (2) nRFSs would exhibit increased ankle dorsiflexion angles and ankle inversion and internal rotation moments and lower GRFs during the early stance phase, and (3) an interaction effect would exist between footstrike patterns and surface conditions on joint moments at the ankle and knee joints.

## Methods

### Participants

Thirty habitual RFSs (age = 24.3 ± 2.4 years, body height = 172.0 ± 6.0 cm, body mass = 67.9 ± 10.5 kg) and thirty habitual nRFSs (age = 28.7 ± 7.1 years; body height = 173.7 ± 4.5 cm; body mass = 67.6 ± 10.2 kg) participated in this study. They ran a minimum of 10 km/week and were recruited through poster information from the Shanghai University of Sport and local runner clubs. All participants were right-leg dominant, as determined by their preferred leg to kick a ball [35]. Only male participants were recruited to eliminate potential gender-related differences in lower extremity biomechanics during running [36]. Participants were excluded if they had any musculoskeletal disorders, cardiovascular disease, or lower extremity surgery within the past 6 months. The recruitment period was from April to May 2020, and all participants signed informed consent forms before the testing.

Prior to the test, the participants were asked to run across a 15-meter runway at their comfortable speed, during which their habitual strike style was identified by strike index (SI). The SI was calculated as the position of the center of pressure (COP) at the initial contact with the ground relative to the long axis of the foot and expressed as a percentage of the full length of the foot [29]. Footstrike patterns were classified based on the SI, where 0–33.3% was defined as RFS and 33.3%–100% was defined as nRFS [14].

### Running surface

Three distinct running surfaces, namely, artificial grass, synthetic rubber, and concrete, were selected because they represent commonly used running terrains and have been employed in numerous previous studies examining the effects of different surfaces on running biomechanics [13, 22, 23]. Each of these customized runways was 15-meter-long and 1-meter-wide. The thickness of synthetic rubber and grass was 2 cm, while the concrete runway was paved using concrete blocks with a thickness of 1 cm. Polyvinyl chloride mattresses (1.6 mm) were placed between the running surface and the floor to minimize friction. These surfaces were identical to those used in our previous studies [13, 22, 23]. The surface stiffness was measured by a ball drop test adopted in accordance with the study of Fu et al. [25]. In particular, a basketball (size 7# with an air pressure of 0.06 MPa) was dropped vertically from a height of 2 meters onto each surface., and the bounce height of the ball was recorded. The coefficient of restitution for each surface was then calculated by normalizing the bounce height to the surface thickness and drop height [36]. The coefficient of restitution for the artificial grass, synthetic rubber, and concrete surfaces was 0.290 ± 0.001, 0.320 ± 0.002, and 0.420 ± 0.010, respectively, confirming that concrete was the stiffest surface, followed by synthetic rubber and artificial grass.

### Data collection

The experiments were conducted at the biomechanics laboratory of the Shanghai University of Sport. First, the participants’ height, weight, and leg length were measured. All of them were then instructed to perform running trails on the three customized indoor runways at a speed of 3.3 m/s, which was randomly assigned for each surface [21]. The participants were allowed to rest for 5 minutes between conditions to avoid accumulating fatigue. The running speed, consistent with previous studies [13, 22], was monitored using a photoelectric timing system (WittySEM, Microgate, Italy). All participants wore the same model of lightweight running shoes (European size 41 to 43, ASICS, SORTIEMAGIC RP 4 TMM467.0790, Japan) to eliminate the shoe thickness and cushioning [37].

Twenty-three retroreflective markers were firmly affixed to the sacrum, superior border of the iliac crest, anterior superior iliac spine, greater trochanter, medial and lateral epicondyles of the femur, medial and lateral ankles, heads of the first and fifth metatarsals, and ends of the second toes and heels of each participant. The markers on the feet were positioned directly on the shoes.

Before data collection, participants were allowed several practice runs to familiarize themselves with the surfaces and ensure they could maintain their habitual footstrike pattern while running at the target speed. During data collection, participants initially stood on a force plate for static calibration trial. They then completed a minimum of five successful running trials, where marker trajectories and synchronized kinetic data were recorded using a ten-camera motion capture system (Nexus, Vicon, Oxford, UK) at 200 Hz, along with the force plate (90 cm × 60 cm; Kistler 9287 C, Winterthur, Switzerland) at 1000 Hz. All the participants were required to complete five successful trials on each surface. A successful running trial was defined as when the subject’s right foot landed within the force plate and the running speed was within 3.3 ± 0.2 m/s.

### Data reduction

Five successful trials were analyzed for each surface condition. For initial processing of the marker trajectories, Vicon Nexus software was used for labelling and gap filling. The marker and GRF data were further processed using Visual 3D software (C-Motion Inc., Rockville, MD, USA), and the lower extremity joint angles were calculated based on the joint coordinate system. Raw marker trajectories and GRF data were filtered using a fourth-order low-pass Butterworth filter with a set cutoff frequency (kinematics: 10 Hz; GRFs: 50 Hz) [38]. GRF data were standardized to participant body weight. Ground contact was defined as the period when the vertical GRF exceeded a threshold of 20 N. All kinematic variables (3D lower extremity joint angles) and kinetic variables (3D GRFs and moment) for the entire stance phase were processed using a custom MATLAB program (MathWorks Inc., Natick, MA). The stance phase were classified into three sub-phases, namely, early (0–33%), mid (34%–67%), and late stance (68%–100%) [39].

### Statistical analysis

A two-way analysis of variance (ANOVA) using SPM was conducted to examine the effects of two footstrike patterns (RFS and nRFS) and three surface conditions (artificial grass, synthetic rubber, and concrete) on the biomechanical variables. The open-source SPM code in MATLAB (www.spm1D.org) was used for the analysis, when significant interactions or main effects were identified, post-hoc SPM analysis with Bonferroni’s correction for multiple comparisons was applied, and paired t-tests were performed to assess specific differences.

## Results

In our study, the SPM analysis was employed to compare the biomechanical parameters throughout the stance phase across different footstrike patterns and surface conditions during running. The results identified specific time intervals within the stance phase where significant differences occurred, with corresponding p-values reported for these ranges. This allowed for detailed insights into how the interaction between footstrike patterns and surface conditions influenced lower extremity biomechanics during running.

### Kinematics

**Fig 1:**
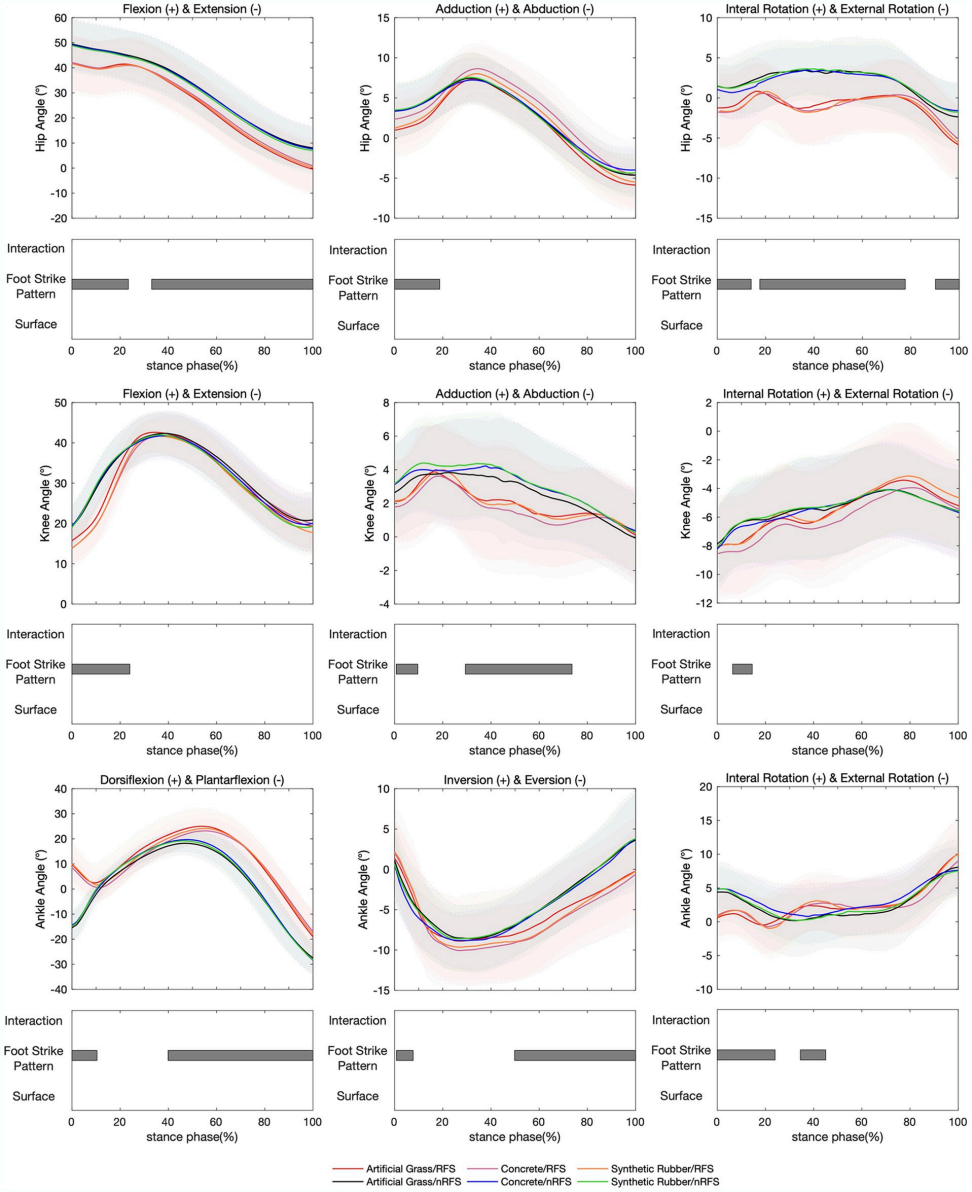
Lower extremity joint angle waveforms of mean and standard deviation over the stance phase of six running conditions. Significant main effects (*p-value*<0.05) for interaction, footstrike pattern, and surface are highlighted for the corresponding time periods analyzed in SPM1d.

No significant interaction was found between footstrike patterns and surface conditions for the joint angles of the hip, knee, and ankle (Fig 1).

In the hip, knee, and ankle joints, no significant differences in joint angles were observed among the runners across the three different overground surfaces (Fig 1).

However, the significant effects of two footstrike patterns on joint angles were observed across various planes of the lower extremity’s three joints during running (Fig 1). At the ankle level, significant differences in dorsiflexion/plantarflexion angles (0–10.4%, F = 7.24, *p-value* = 0.041; 39.9%–100%, F = 7.24, *p-value*<0.001), inversion/eversion angles (0.8%–7.7%, F = 7.35, *p-value* = 0.045; 49.8%–100%, F = 7.35, *p-value*<0.001), and internal rotation/external rotation angles (0–24.0%, F = 7.52, *p-value* = 0.011; 34.5%–45.0%, F = 7.52, *p-value* = 0.040) were found between RFSs and nRFSs. The results demonstrated that RFSs exhibited greater dorsiflexion angles, smaller eversion and internal rotation angles during early stance, and larger eversion and internal rotation angles during mid-to-late stance compared with nRFSs (*p-values*<0.05). At the knee level, significant differences were observed in flexion/extension angles (0–24.0%, F = 7.03, *p-value* = 0.011), adduction/abduction angles (0.7%–9.6%, F = 6.90, *p-value* = 0.046; 29.4%–73.6%, F = 6.90, *p-value* = 0.004), and internal rotation/external rotation angles (6.4%–14.5%, F = 6.63, *p-value* = 0.048). The results indicated that RFSs exhibited reduced knee flexion, adduction, and external rotation angles during mid-to-early stance. At the hip level, significant differences were found in flexion/extension angles (0–23.4%, F = 5.73, *p-value* = 0.044; 33.1%–100%, F = 5.73, *p-value* = 0.018), adduction/abduction angles (0–18.7%, F = 6.82, *p-value* = 0.034), and internal rotation/external rotation angles (0–14.0%, F = 7.43, *p-value* = 0.033; 17.6%–77.7%, F = 7.43, *p-value*<0.001; 90.2%–100%, F = 7.43, *p-value* = 0.040), which indicated that RFSs exhibited smaller flexion, adduction, and internal rotation angles compared with nRFSs (*p-values*<0.05).

### Kinetics

#### Ground reaction forces

**Fig 2:**
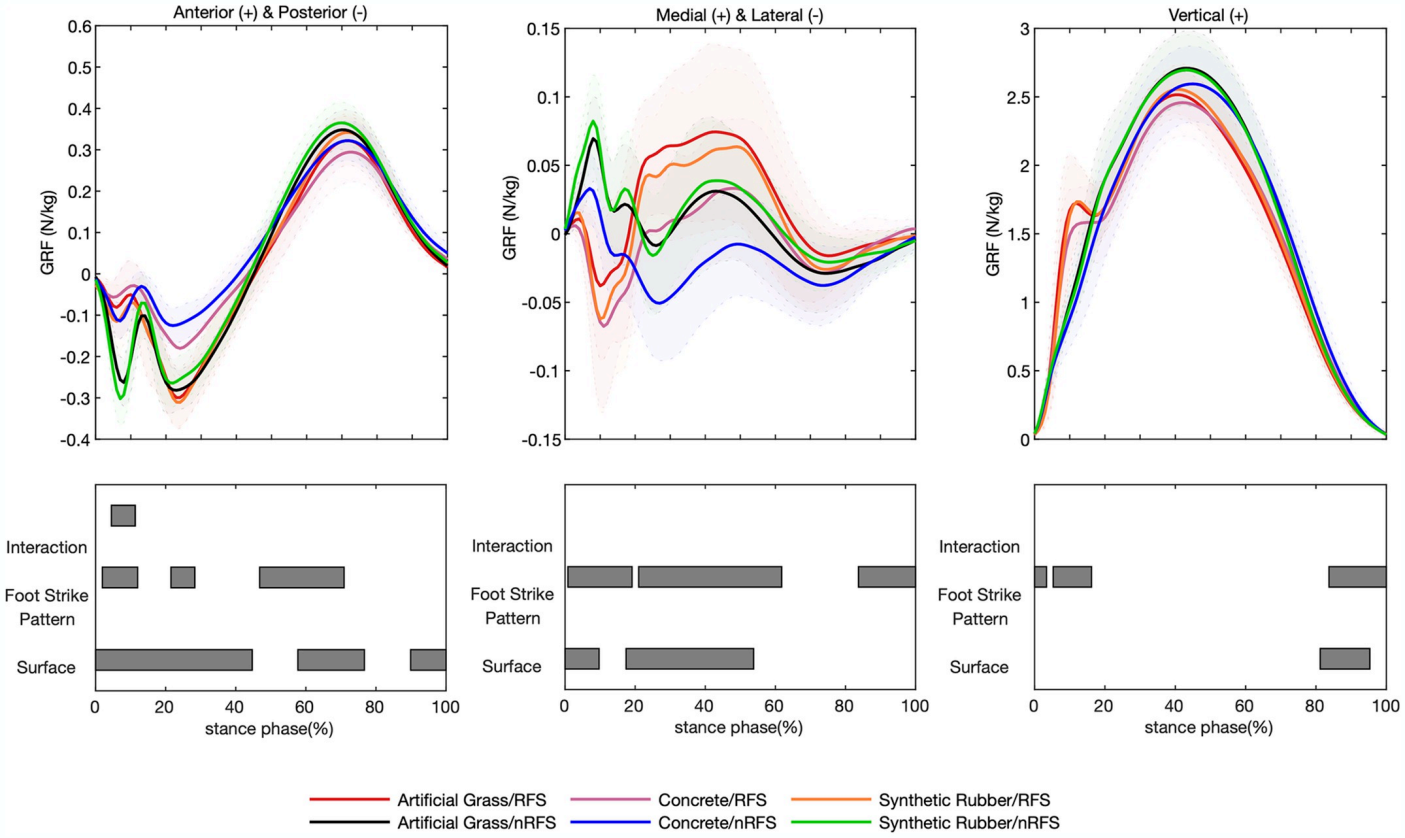
Ground reaction force waveforms of mean and standard deviation over the stance phase of six running conditions. Significant main effects (*p-value*<0.05) for interaction, footstrike pattern, and surface are highlighted for the corresponding time periods analyzed in SPM1d.

The interaction effect between overground surfaces and footstrike patterns of GRF was only evident in the anterior-posterior GRF (4.5%–11.3%, F = 6.44, *p-value* = 0.019) (Fig 2). Post-hoc analysis showed that participants with the same footstrike pattern exhibited greater posterior GRF when running on softer surfaces (*p-values*<0.05). The nRFSs had higher posterior GRF across different surfaces compared to the RFSs running on the corresponding surfaces (*p-values*<0.05). Among them, the nRFSs had the highest posterior GRF when running on synthetic rubber.

During running on three different surfaces (concrete, artificial grass, and synthetic rubber), significant differences in GRF were observed across all three planes (Fig 2): anterior-posterior GRF (0–44.7%, F = 6.40, *p-value*<0.001; 57.7%–76.7%, F = 6.40, *p-value*<0.001; 89.9%–100%, F = 6.40, *p-value* = 0.003), medial-lateral GRF (0–9.7%, F = 6.43, *p-value* = 0.019; 17.4%–53.8%, F = 6.43, *p-value*<0.001), and vertical GRF (81.2%–95.3%, F = 6.59, *p-value*<0.001). Post-hoc analysis revealed that running on softer overground surfaces induced greater posterior GRF during 0–44.7% and 89.9%–100% of the stance and anterior GRF during 57.7%–76.7% of the stance (*p-values*<0.05). Meanwhile, running on surfaces with lower stiffness caused greater medial GRF during midstance and reduced vertical GRF during late stance (*p-values*<0.05).

Our research findings demonstrated significant differences in GRF across the three planes for runners employing two distinct footstrike patterns (Fig 2): anterior-posterior GRF (1.9%–12.0%, F = 9.61, *p-value* = 0.004; 21.5%–28.5%, F = 9.61, *p-value* = 0.017; 46.8%–70.9%, F = 9.61, *p-value*<0.001), medial-lateral GRF (0.8%–19.1%, F = 9.51, *p-value*<0.001; 21.0%–61.8%, F = 9.51, *p-value*<0.001; 83.7%–100%, F = 9.51, *p-value*<0.001), and vertical GRF (0–3.4%, F = 9.89, *p-value* = 0.033; 5.3%–16.2%, F = 9.89, *p-value* = 0.001; 19.4%–80.7%, F = 9.89, *p-value*<0.001). These results revealed that compared to nRFSs, RFSs exhibited reduced posterior GRF during early stance, transitioning to decreased anterior GRF during mid-to-late stance (*p-values*<0.05). Meanwhile, nRFSs demonstrated increased medial GRF during midstance, and their lateral GRF at late stance exceeded that of RFSs (*p-values*<0.05). Compared with that of nRFSs, the vertical GRF of RFSs was significantly smaller at 0–3.4% and 19.4%–80.4% of the support phase but greater at 5.3%–16.2% (*p-values*<0.05).

#### Lower extremity joint moment

**Fig 3:**
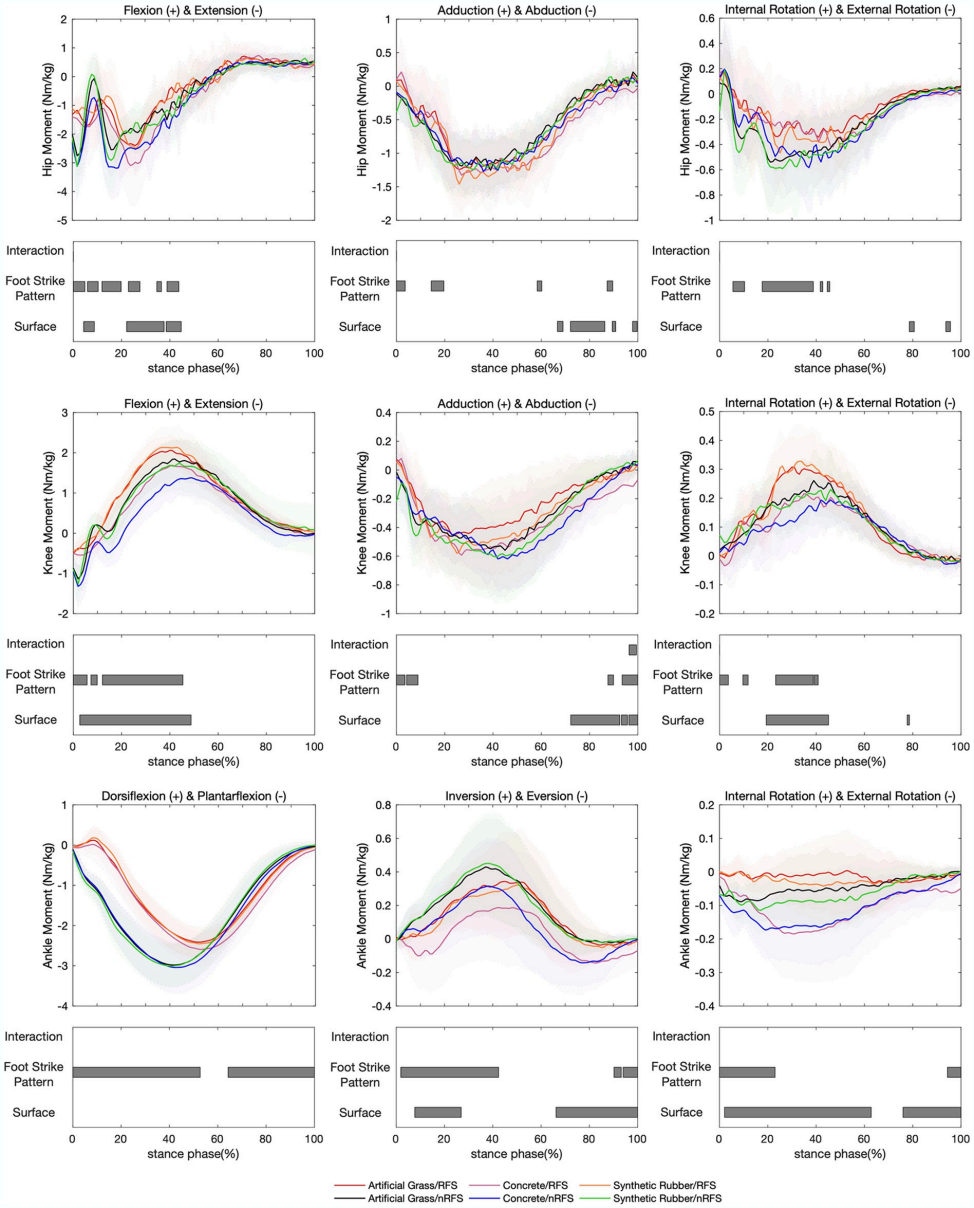
Lower extremity joint moment waveforms of mean and standard deviation over the stance phase of six running conditions. Significant main effects (*p-value*<0.05) for interaction, footstrike pattern, and surface are highlighted for the corresponding time periods analyzed in SPM1d.

The interaction effect of joint moment was observed solely in the knee joint’s adduction—abduction moment (96.4%–99.4%, F = 7.30, *p-value* = 0.021) (Fig 3). Post-hoc analysis indicated that compared to other footstrike patterns and surface conditions, RFSs exhibited the greatest knee abduction moment when running on concrete (*p-value* = 0.013).

During running on three different overground surfaces, significant differences in joint moments were observed across all three joints (Fig 3). At the ankle, significant differences were observed in inversion/eversion moments (7.7%–26.9%, F = 6.51, *p-value*<0.001; 66.2%–100%, F = 6.51, *p-value*<0.001) and internal rotation/external rotation moments (2.1%–62.8%, F = 6.85, *p-value*<0.001; 76.0%–100%, F = 6.85, *p-value*<0.001). Post-hoc analysis revealed that during early and late stances, running on the artificial grass surface (the softest) induced greater inversion moments at the ankle compared with stiffer surfaces (*p-value*<0.001). Moreover, the internal rotation moments at the ankle joint increased as surface stiffness decreased during most of the stance phase (*p-values*<0.05). At the knee, significant differences were found among overground surfaces in flexion/extension moments (2.7%–48.8%, F = 6.88, *p-value*<0.001), adduction/abduction moments (72.2%–92.5%, F = 7.30, *p-value*<0.001; 93.2%–95.7%, F = 7.30, *p-value* = 0.021; 96.3%–100%, F = 7.30, *p-value* = 0.007), and internal rotation/external rotation moments (19.3%–45.1%, F = 7.61, *p-value*<0.001; 77.7%–78.6%, F = 7.61, *p-value* = 0.043). Post-hoc analysis indicated that, with increasing surface stiffnesses, knee extension and internal rotation moments increased during midstance, along with greater knee abduction and external rotation moments during late stance (*p-values*<0.05). At the hip, the significant main effects of surface conditions on flexion/extension moments (4.3%–8.7%, F = 8.02, *p-value*<0.001; 22.1%–37.6%, F = 8.02, *p-value*<0.001; 38.5%–44.7%, F = 8.02, *p-value*<0.001), adduction/abduction moments (66.7%–69.0%, F = 8.02, *p-value* = 0.004; 72.1%–86.3%, F = 8.02, *p-value*<0.001; 89.4%–90.8%, F = 8.02, *p-value* = 0.020; 97.8%–99.7%, F = 8.02, *p-value* = 0.012), and internal rotation/external rotation moments (78.6%–80.6%, F = 7.82, *p-value* = 0.019; 93.7%–95.6%, F = 7.82, *p-value* = 0.022) were observed. Post-hoc analysis revealed that stiffer surfaces were associated with increased hip extension moments during early stance and increased hip abduction and external rotation moments during late stance (*p-values*<0.05).

We also observed the significant effects of two footstrike patterns on joint moments of the lower extremity’s three joints across the three planes during running (Fig 3). At the ankle level, significant differences in dorsiflexion/plantarflexion moments (0–52.7%, F = 8.95, *p-value*<0.001; 64.3%–100%, F = 8.95, *p-value*<0.001), inversion/eversion moments (1.9%–42.4%, F = 9.75, *p-value*<0.001; 90.2%–93.2%, F = 9.75, *p-value* = 0.041; 94.0%–100%, F = 9.75, *p-value* = 0.017), and internal rotation/external rotation moments (0–23.0%, F = 10.34, *p-value*<0.001; 94.4%–100%, F = 10.34, *p-value* = 0.010) between RFSs and nRFSs were demonstrated. These results revealed that RFSs exhibited greater dorsiflexion, eversion, and internal rotation moments during early stance and greater plantarflexion, eversion, and external rotation moments during late stance (*p-values*<0.05). At the knee level, significant differences in flexion/extension moments (0–5.7%, F = 10.34, *p-value* = 0.008; 7.3%–9.9%, F = 10.34, *p-value* = 0.035; 12.1%–45.4%, F = 10.34, *p-value*<0.001), adduction/abduction moments (0–3.5%, F = 11.15, *p-value* = 0.010; 4.2%–8.9%, F = 11.15, *p-value* = 0.004; 87.9%–89.9%, F = 11.15, *p-value* = 0.03; 93.5%–100%, F = 11.15, *p-value*<0.001), and internal rotation/external rotation moments (0–3.6%, F = 11.70, *p-value* = 0.003; 9.6%–11.8%, F = 11.70, *p-value* = 0.016; 23.2%–38.8%, F = 11.70, *p-value*<0.001; 39.2%–40.8%, F = 11.70, *p-value* = 0.031) were found between the two footstrike patterns. In the early-mid stance, the knee extension and internal rotation moments in RFSs were generally smaller than those in nRFSs (*p-values*<0.05). Furthermore, the results in the coronal plane of the knee joint demonstrated that RFSs exhibited greater adduction angle during early stance but smaller adduction angle during late stance compared with nRFSs (*p-values*<0.05). At the hip level, we observed significant differences in flexion/extension moments (0–4.8%, F = 12.44, *p-value*<0.001; 5.9%–10.3%, F = 12.44, *p-value*<0.001; 11.9%–19.8%, F = 12.44, *p-value*<0.001; 22.8%–27.6%, F = 12.44, *p-value*<0.001; 34.6%–36.5%, F = 12.44, *p-value* = 0.017; 38.8%–43.7%, F = 12.44, *p-value*<0.001), adduction/abduction moments (0–3.6%, F = 12.45, *p-value*<0.001; 14.4%–19.6%, F = 12.45, *p-value*<0.001; 58.3%–60.2%, F = 12.45, *p-value* = 0.012; 87.3%–89.6%, F = 12.45, *p-value* = 0.006), and internal rotation/external rotation moments (5.5%–10.3%, F = 12.10, *p-value*<0.001; 17.6%–38.8%, F = 12.10, *p-value*<0.001; 41.7%–42.7%, F = 12.10, *p-value* = 0.041; 44.6%–45.6%, F = 12.10, *p-value* = 0.042) between the footstrike patterns. These significant differences revealed that hip flexion/extension moments exhibited significant fluctuations throughout the entire stance phase, with RFSs showing smaller extension moments than nRFSs at 0–4.8%, 11.9%–19.8%, 34.6%–36.5%, and 38.8%–43.7% intervals (*p-values*<0.05). Meanwhile, nRFSs exhibited smaller hip extension moments in the remaining significant intervals (*p-values*<0.05). RFSs demonstrated greater adduction moments during early stance and greater abduction moments during midstance (*p-values*<0.05). However, the hip external rotation moments were lower in RFSs than nRFSs during the early-mid stance (*p-values*<0.05).

## Discussion

To the best of our knowledge, this study represents the first attempt to utilize SPM to analyze the effects of footstrike patterns and running surfaces on lower extremity biomechanics during running. SPM analysis allowed for a comprehensive examination of continuous biomechanical data, revealing the intricate relationship between footstrike patterns and surface stiffness in running mechanics. While the results partially corroborate our initial hypothesis regarding the effect of surface stiffness on lower extremity biomechanics, they revealed an increase in both medial and vertical GRFs on stiffer surfaces, with no significant changes observed in joint angles. Notably, running on softer surfaces led to increased ankle inversion and internal rotation moments throughout most of the stance phase. Furthermore, there were increases in knee and hip flexion moments during early stance, and reductions in knee abduction and hip abduction and external rotation moments in late stance. These findings underscore the importance of considering running surface conditions in the design of training programs and assessment of the risk of musculoskeletal injuries in runners.

In our study results, the data concerning different footstrike patterns partially supported the hypothesis. During the early stance, regardless of surface type, RFSs exhibited significantly greater dorsiflexion and smaller inversion and internal rotation angles at the ankle joint compared to nRFSs. In the mid-to-late stance phase, RFSs demonstrated greater dorsiflexion, inversion, and internal rotation angles than nRFSs. RFSs also demonstrated reduced extension, adduction, and internal rotation angles at the knee and hip joints. In addition, distinct GRF patterns were noted between footstrike types, with RFSs exhibiting higher vertical GRF at initial foot contact, which gradually diminished to level below those of nRFSs. In terms of moments, nRFSs displayed greater dorsiflexion, inversion, and external rotation moments at the ankle joint during most of the midstance, while RFSs experienced greater flexion, adduction, and internal rotation moments at the knee joint.

Our results partially support the hypothesis regarding the interaction between footstrike patterns and overground conditions. We observed this interaction effect only in the anterior-posterior GRF during early stance and the knee flexion-extension moment at the end of stance. This finding underscores the complexity of the relationship between these two factors during running. The interaction effect suggests that the combined influence of footstrike patterns and surface stiffness may alter the load transfer mechanisms, presenting distinct challenges to the knee joint, and thus may offer valuable insights for injury prevention.

### Kinematics

Our results showed no significant influence of surface type on lower extremity joint angles for either RFSs or nRFSs, aligning with Dixon et al. [16], who found minimal kinematic variations across different surfaces. By contrast, Hardin et al. [40] indicated that increased surface stiffness led to decreased hip and knee flexion at initial contact and maximum hip flexion. Shen et al. [8] reported that the peak hip flexion angle was greater after long-distance running on asphalt roads (stiff) compared to that after running on other surfaces (soft). The observed variations between studies may be attributed to differences in surface stiffness and gradient settings across the experimental trials on various overground surfaces. Additionally, nRFSs may mitigate impact through specific foot structures, such as the foot arch and the plantar fat pad of the forefoot [41], where RFSs likely rely on heel cushioning. Despite these differing impact absorption strategies, habitual footstrike patterns may lead to similar movement strategies across different surfaces.

In this study, the influence of footstrike patterns on lower extremity joint angles was substantial, affecting nearly every joint in all planes across all running surfaces. RFSs and nRFSs represent distinct biomechanical adaptations, with nRFSs demonstrating greater plantar flexion angle at the ankle at initial contact, allowing the forefoot to make contact with the ground first. This necessitates greater angle of knee flexion and hip flexion to accommodate the footstrike. Consistent with our findings, Thompson et al. [29] also reported significant differences in lower extremity kinematics between RFSs and nRFSs under shod conditions. During the mid-to-late stance phase, nRFS runners exhibited greater dorsiflexion and inversion angles at the ankle, which is consistent with previous studies that found variations in ankle eversion angles between different footstrike patterns [27, 40, 42]. RFS typically shows larger ankle eversion angles, likely due to the prolonged ground contact time associated with this pattern [43], suggesting that the RFS may rely more on coronal plane movements of the ankle joint to maintain stability.

Furthermore, we propose that during the mid-to-late stance, runners experience a forward shift in their COP, which alters the direction and magnitude of the GRF. As a result, the ankle joint must adjust its angle to accommodate these forces. During this phase, particularly during propulsion, the calf muscles (gastrocnemius and soleus) engage more actively to generate propulsive force, leading to increase in the dorsiflexion and inversion angles at the ankle joint, facilitating a more effective takeoff from the ground [44, 45]. Given that nRFS involves initial forefoot contact, the greater plantarflexion and inversion angles at the ankle during the mid-to-late stance phase may increase the risk of lower extremity stress-related injuries (such as metatarsal stress fractures) and soft tissue injuries (such as Achilles tendinopathy and plantar fasciitis), as these angles result in greater loading of the plantar flexors and Achilles tendon compared to RFS [30, 46].

### Kinetics

#### Ground reaction forces

In this study, we observed the influences of different running surfaces on GRF. Previous studies have indicated that, without distinguishing between footstrike patterns, medial GRF increases as ground stiffness decreases [31, 42]. Our findings align with this, as we observed greater medial GRF on soft surfaces during midstance. This is possibly due to the instability of the soft ground, which impairs dynamic postural stability and necessitates higher medial-lateral GRF to maintain balance. Toward the late stance, we observed higher vertical GRF on hard surfaces, consistent with the findings of Kerdok et al. [43] and Ferris et al. [47]. This may be due to the reduced energy absorption by hard surfaces compared to soft ones, requiring runners to exert more force for propulsion as less energy is returned to the body.

Our research findings also reveal variations in the magnitudes of GRF across different directions for both footstrike patterns throughout the entire stance phase across all running surfaces. Notably, nRFSs exhibited greater anterior and vertical GRFs compared to RFSs, with statistical significance observed during the mid-to-late stance phase. While many studies have reported lower vertical GRF for nRFSs during running compared to RFSs [30, 41, 48], Valenzuela et al. [31] found significantly greater peak vertical GRF under nRFS conditions than under RFS conditions. One potential explanation for this discrepancy is that Valenzuela’s study allowed participants to run at their preferred pace, and RFSs ran at higher speeds than nRFSs, which can influence vertical GRFs [49]. Furthermore, the RFSs in our study exhibited greater ankle dorsiflexion angle compared to nRFSs. Previous studies have indicated that an increased ankle dorsiflexion angle is associated with reduced vertical GRF [50], suggesting that nRFSs would exhibit lower vertical GRF. This finding, however, contradicted the results of our current study. In terms of medial-lateral GRF, our results show that RFSs exhibited larger lateral GRF during the early stance and lower lateral GRF towards the end of stance compared to nRFSs. This could be explained by the mechanics of rearfoot striking. RFSs initially contact the ground with the rearfoot, followed by the downward rolling motion of the forefoot, which applies inward pressure from the lateral side of the foot onto the ground. This inward pressure leads to a corresponding external GRF exerted by the ground on the body, leading to a greater lateral GRF in early stance for RFSs. In contrast, during the late stance, nRFSs transition from initial contact at the first metatarsophalangeal joint to full forefoot contact. As the force application shifts medially, nRFSs exhibit a larger lateral GRF during the late stance.

#### Lower extremity joint moment

Our research results highlighted the significant influence of surface conditions on lower extremity joint moments in all runners. Running on stiffer surfaces leads to greater extension, external rotation, and internal rotation moments at the hip joint within all significant intervals, indicating increased muscle activation and recruitment around the hip joint to maintain dynamic postural stability. Therefore, the hip may play a crucial role in controlling and adjusting body movements when running on stiffer surfaces. Higher activation of the hip muscles would help better control the transmission of GRF, thereby reducing the impact on the knee and ankle joints. Conversely, insufficient or uncoordinated hip activation might increase the risk of knee or other lower extremity injuries.

Our experimental findings demonstrated a significant increase in inversion and internal rotation moments at the ankle joint when running on soft surfaces, persisting for most of the stance phase. This result suggests that runners rely heavily on the ankle joint for balance control when running on soft ground. However, running on stiffer surfaces reduces impact cushioning at the ankle joint, leading to greater force transmission to the knee and even the hip joint. Supporting this, Yamin et al. [15] reported larger lower extremity joint moments and lower impact absorption when running on artificial grass (hard) compared with those when running on rubber (soft). Dragoo et al. [51] also found more severe anterior cruciate ligament injuries when running on artificial grass than running on natural grass. These findings underscore the importance of surface selection in minimizing injury risks during running.

In the context of all running surface conditions, we identified significant differences in joint moments among the ankle, knee, and hip joints between the two footstrike patterns. At the ankle joint level, nRFS exhibited smaller eversion and external rotation moments during the mid-to-late stance compared to RFS, consistent with Valenzuela et al. [31], who reported lower external rotation moments during this phase in nRFSs. Similarly, Kulmala et al. [30] found that nRFSs exhibited a greater peak internal rotation moment, likely attributed to the substantial divergence in the dorsiflexion—plantarflexion angles of the ankle joint when adopting distinct footstrike patterns. We propose that, compared to RFSs, nRFSs may facilitate an earlier shift of the COP toward the forefoot during propulsion [41], optimizing force transmission and reducing the demand for eversion and external rotation moments. Additionally, nRFSs primarily rely on the forefoot and calf muscles for force generation [30], which may result in greater muscle coordination. This effective activation of the muscles can minimize unnecessary eversion and external rotation moments, promoting enhanced stability control of the lower extremities. The nuanced interplay of these biomechanical factors elucidates the intricate distinctions in ankle joint mechanics between the two footstrike patterns. In this context, the present study delved into the subtle nuances of ankle joint biomechanics, providing profound insights into the impact of footstrike pattern selection on joint mechanics.

Regarding the knee, Kulmala et al. [30] observed lower knee abduction moments in nRFSs, which our study corroborates, as nRFSs exhibited smaller knee abduction moments during the late stance phase compared to RFSs. This finding aligned with the notion that variations in knee joint moments and ground contact dynamics in nRFS may influence the overall external forces acting on the limb during gait. RFSs predominantly utilize the fat pad of their rearfoot for cushioning, and nRFSs rely on the Achilles tendon for shock absorption [41]. In conjunction with the experimental outcomes of GRF, our findings suggested that the dissimilarities in cushioning capacity between these two patterns might contribute to variations in GRF. Furthermore, such cushioning patterns indicated distinct injury-prone sites in the lower extremities for runners with different footstrike patterns. In particular, the high incidence of ankle joint injuries in nRFSs might be associated with the large plantarflexion angle at initial ground contact and the transmission of impact forces to the ankle joint through the forefoot [52]. By contrast, RFSs might be predisposed to knee joint injuries because the knee joint absorbs a great magnitude of impact forces [33, 53]. Kumala et al. [30] demonstrated in their study comparing two footstrike patterns that nRFSs exhibit lower knee frontal plane moments compared to RFSs. Previous research has shown that higher knee frontal plane moments correlate with increased medial compartment knee loading [54, 55], often associated with degenerative knee conditions like medial tibiofemoral osteoarthritis [56]. This conclusion highlights distinct lower extremity injury patterns associated with different landing patterns among runners, substantiated by biomechanical evidence. This elucidation contributes to a comprehensive understanding of the biomechanical factors influencing the external forces and moments in different footstrike patterns.

At the hip joint, nRFSs exhibited larger abduction and external rotation moments during early stance, indicating increased activation of the hip abductor muscles to maintain balance in the initial support phase. Although our results showed similar trends in hip flexion/extension moments for both footstrike patterns during early stance, nRFSs demonstrated a steeper change in slope than RFSs, suggesting more pronounced moment variations. This could be due to the relatively lower hip joint stiffness in nRFSs compared to RFSs, which may exhibit greater hip joint stiffness during running, particularly at the initial ground contact, to mitigate the impact forces transmitted through the hip joint due to the lack of ankle joint cushioning. This phenomenon could lead to small magnitude changes in moments. However, the role of hip joint stiffness in different footstrike patterns remains understudied, warranting further investigation in future research.

### Limitations

This study has several limitations that should be considered. Firstly, Phinyomark et al. [36] and Dufek et al. [57] showed significant sex differences in kinematic and kinetic variables during running. However, our study only recruited male runners, which may limit the generalizability of our findings to female runners. Future studies should aim to include both genders to provide a more comprehensive understanding of these biomechanical variables. Secondly, the experiment was conducted on a relatively short track (15-meter), possibly limiting the applicability of our findings to long-distance running scenarios. Longer monitoring periods and track lengths in future research could yield more relevant insights for endurance running. Thirdly, our experiment lacked the calculation and comparison of spatiotemporal parameters, particularly contact time, which could have provided additional insights into our results. Future research could focus on comparing the spatiotemporal parameters of runners with different footstrike patterns on various surfaces, providing deeper theoretical insights into gait mechanics.

## Conclusion

Our findings underscore the influence of footstrike patterns and surface conditions on the kinematics and kinetics of lower extremity joints. Using SPM analysis, we elucidated how biomechanical variables differ across specific phases of the stance period. Our study revealed that nRFSs exhibited a greater ankle inversion angle and higher inversion and internal rotation moments during the mid-to-late stance compared to RFSs. This finding indicated that nRFSs rely more heavily on ankle joint strategies for control, which may increase their risk of ankle joint injuries. Meanwhile, RFSs demonstrated a greater knee abduction moment during the late stance. This finding suggests that RFSs subject their knee joint to high stress, emphasizing their need to pay attention to their knee joint health. Additionally, we found that running on stiffer surfaces is correlated with higher vertical GRF, signifying increased impact forces on the body. Therefore, regardless of footstrike pattern, runners may benefit from selecting softer surfaces to mitigate injury risk from impact.

## Supporting information

S1 Data Raw data of main figures used in this study. (ZIP)
